# Land Use Affects Carbon Sources to the Pelagic Food Web in a Small Boreal Lake

**DOI:** 10.1371/journal.pone.0159900

**Published:** 2016-08-03

**Authors:** Päivi Rinta, Maarten van Hardenbroek, Roger I. Jones, Paula Kankaala, Fabian Rey, Sönke Szidat, Matthew J. Wooller, Oliver Heiri

**Affiliations:** 1 Institute of Plant Sciences, University of Bern, Bern, Switzerland; 2 Oeschger Centre for Climate Change Research, University of Bern, Bern, Switzerland; 3 Department of Biological and Environmental Science, University of Jyväskylä, Jyväskylä, Finland; 4 Department of Environmental and Biological Sciences, University of Eastern Finland, Joensuu, Finland; 5 Department of Chemistry and Biochemistry, University of Bern, Bern, Switzerland; 6 School of Fisheries and Ocean Sciences, University of Alaska, Fairbanks, Alaska, United States of America; 7 Water and Environmental Research Center, University of Alaska, Fairbanks, Alaska, United States of America; GEOMAR Helmholtz Center for Ocean Research, GERMANY

## Abstract

Small humic forest lakes often have high contributions of methane-derived carbon in their food webs but little is known about the temporal stability of this carbon pathway and how it responds to environmental changes on longer time scales. We reconstructed past variations in the contribution of methanogenic carbon in the pelagic food web of a small boreal lake in Finland by analyzing the stable carbon isotopic composition (δ^13^C values) of chitinous fossils of planktivorous invertebrates in sediments from the lake. The δ^13^C values of zooplankton remains show several marked shifts (approx. 10 ‰), consistent with changes in the proportional contribution of carbon from methane-oxidizing bacteria in zooplankton diets. The results indicate that the lake only recently (1950s) obtained its present state with a high contribution of methanogenic carbon to the pelagic food web. A comparison with historical and palaeobotanical evidence indicates that this most recent shift coincided with agricultural land-use changes and forestation of the lake catchment and implies that earlier shifts may also have been related to changes in forest and land use. Our study demonstrates the sensitivity of the carbon cycle in small forest lakes to external forcing and that the effects of past changes in local land use on lacustrine carbon cycling have to be taken into account when defining environmental and ecological reference conditions in boreal headwater lakes.

## Introduction

The food web of small, boreal forest lakes can be characterized by high contributions of carbon from methane (CH_4_) produced by decomposition of organic matter in anoxic lake sediments and water columns [1]. Humic water with strong light absorption and significant input of terrestrial organic matter often leads to steep stratification and hypolimnetic anoxia creating favorable conditions for methanogenesis [2]. Methanogenic carbon is incorporated into biomass by methane-oxidizing bacteria (MOB) [3], which can be consumed by benthic and pelagic invertebrates [4,5] and hence represent a potential carbon and energy source for the aquatic food web. Studies of some small lakes in boreal Finland have shown that methanogenic carbon can contribute more than half of the carbon incorporated by chironomid larvae [6] or zooplankton [5,7]. Similarly large contributions of methanogenic carbon to food webs can be expected in many other forest lakes of similar size, since small and humic lakes with high hypolimnetic CH_4_ accumulation rates are very common in the boreal zone [8].

Many boreal headwater catchments have experienced major changes in vegetation and land use during past decades. For example, in Finland the intensification of agriculture and forestry has led to abandonment of hay meadows and pastures in remote forest areas [9] and to draining of half of the country’s mires since the 1950s [10]. Understanding how lacustrine carbon cycling has responded to recent environmental changes is of relevance for establishing natural reference conditions for lakes, as presently required by the European Water Framework Directive (WFD) [11], as well as for predicting how lakes and their carbon cycles will respond to future environmental change. A number of studies have described the effects of environmental changes on lacustrine carbon cycling in boreal lakes over several years [12,13]. However, very little is known about longer term variations in carbon cycling in these lakes and their response to external pressures on time scales in which changes in vegetation cover and land-use practices take place.

Contributions of different carbon sources to aquatic food webs can be investigated using stable carbon isotope techniques [14–16]. Contributions by methanogenic carbon are particularly apparent due to the distinctly more negative carbon isotopic values (δ^13^C values) of CH_4_ (-80 to -50 ‰) [17] and of MOB (-100 to -55 ‰) [18] compared to photoautotrophically produced organic carbon (-35 to -8 ‰) [19–22]. Changes in the proportional contribution of these different carbon sources are reflected in the δ^13^C values of many planktonic and benthic invertebrates and of their fossilizing chitinous structures, such as resting stages of Cladocera (ephippia) or freshwater Bryozoa (statoblasts) [23,24]. Some aspects of the past carbon cycling of lakes can therefore be examined by analyzing the δ^13^C values of these fossil structures isolated from dated lake sediments [25–28]. δ^13^C analyses of bulk sedimentary organic matter provide an integrated estimate of changes in the carbon isotopic composition of organic matter deposited in a lake from lacustrine sources as well as from the lake catchment and can be strongly affected by diagenetic processes within sediments. In contrast, taxon-specific δ^13^C analyses of chitinous invertebrate remains can, due to the specific habitat and feeding ecology of certain invertebrate groups, record changes that cannot be distinguished in bulk sediment records [28,29]. Furthermore, in lake sediment records chitinous invertebrate fossils are less susceptible to modification of their chemical and isotopic composition than bulk organic matter [30].

Here we present a reconstruction of δ^13^C values of planktivorous invertebrates in Lake Mekkojärvi in southern Finland, which has exceptionally high contributions of methanogenic carbon in the modern pelagic food web. We focus our analyses on ephippia of *Daphnia* (Cladocera) and statoblasts of *Plumatella* (Bryozoa), which are abundant in the sediments from the lake and originate from organisms known to feed on organic particles in the water column [31,32] potentially including MOB. Our main aim is to assess whether the δ^13^C values of *Daphnia* presently found in Mekkojärvi are representative of the values during the past centuries or whether they record major changes, which could indicate changes in the proportional contribution by methanogenic carbon to the pelagic food web. In addition, we assess if these changes coincided with evidence for human-induced land-use changes around Mekkojärvi. This assessment of the present carbon cycle of the lake in relation to its longer-term development will improve understanding of the multi-decadal variability in carbon cycling in small lakes and of the stability of contributions of different carbon sources to their food webs under changing environmental conditions. These are important issues in the context of the assessment of the natural reference state of small forest lakes with high contributions of methanogenic carbon to their food webs, since changes in the predominant carbon source may affect the response of these ecosystems and their biotic communities to external pressures, such as eutrophication or climate change [33,34].

## Material and Methods

### Study site

**Fig 1:**
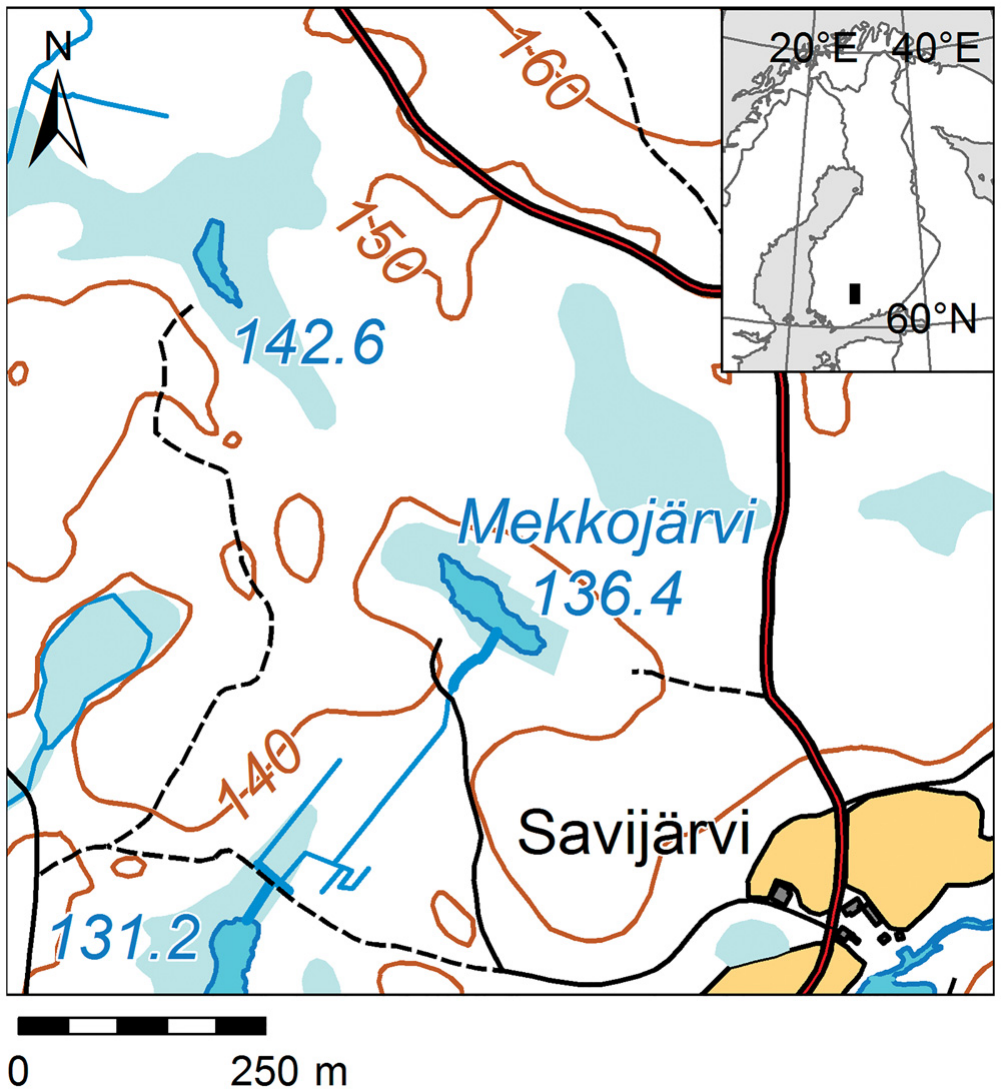
Geographical location of Lake Mekkojärvi and its catchment area. Data source: National Land Survey of Finland, Topographic database, 8/2015. Licensed under Creative Commons (CC) Attribution 4.0 International License.

Mekkojärvi is a small (surface area 0.3 ha, max. depth 4 m), sheltered, polyhumic lake situated in the Evo forest area in southern Finland (61.23°N, 25.14°E; Fig 1). Due to anoxia under winter ice cover, the lake does not support planktivorous fish and the zooplankton community is dominated by abundant large-bodied *Daphnia longispina* [35]. Mekkojärvi has been the subject of many contemporary limnological and ecological studies [35,36]. Several carbon addition experiments [7,37] and studies analyzing the δ^13^C values of zooplankton [5,38] have revealed that a large proportion of the carbon presently incorporated by zooplankton originates from methanogenesis, indicated by δ^13^C values from *Daphnia* that are more negative than -50 ‰ and proportional contributions of MOB estimated to be up to 50% of *Daphnia* diets during the autumnal overturn [7,37].

### Sediment core

A sediment core (78 cm length) was obtained from Mekkojärvi with a gravity corer (Uwitec, Austria) from the deepest point of the lake in April 2011. No specific permissions were required for accessing the lake and collecting sediment under the Finnish Everyman’s Right. The field studies did not involve endangered or protected species. The core was subsampled in the field at 1 cm resolution. Due to the high water content in the uppermost part of the core, the top 2 cm were subsampled together. All sediment samples were freeze dried and stored dry and dark until analysis. Gamma spectrometry for sediment dating was conducted at the Department of Chemistry and Biochemistry at the University of Bern (Switzerland). Approximately 0.8 g of freeze-dried sediment was analyzed for ^210^Pb (46.5 keV), ^226^Ra (351.9 and 609.3 keV), and ^137^Cs (661.7 keV) for approximately 30 hours for the uppermost 5 cm and approximately 100 hours for the lower part of the core using a Broad Energy Germanium (BEGe) detector (Canberra, Germany) with low background and high absolute full-energy peak efficiencies for close on-top geometries (> 20% and approx. 5% for ^210^Pb and ^137^Cs, respectively). Age-depth modelling using ^210^Pb was based on a constant rate of supply (CRS) model [39–41], but no reference points from the ^137^Cs activity were used due to the unusual activity profile (see Results). A series of five terrestrial plant macrofossils from the depth of 45 cm downwards were measured for radiocarbon dates using accelerator mass spectrometry (AMS) at the Poznań Radiocarbon Laboratory (Poland). However, these radiocarbon dates were not used to constrain the age of the record due to their unrealistically old and variable dates (see Results). The lowest section of the core therefore, remained undated and we focused our further analyses only on the uppermost 30 cm. Since ^137^Cs measurements produced unexpected results, a second series of six ^14^C dates were measured using MICADAS AMS at the Laboratory for the Analysis of Radiocarbon with AMS at University of Bern (Switzerland) [42] from terrestrial plant remains from the depths from 6 to 12 cm and calibrated using the IntCal13 calibration curve with the NH1 extension [43,44]. Samples were selected to confirm the expected location of the bomb peak in the ^137^Cs profile. This approach relies on the rapid increases of both ^137^Cs and atmospheric ^14^C as a consequence of above-ground nuclear bomb testing [45].

For analyzing carbon and nitrogen content and δ^13^C values of sedimentary organic matter (SOM), subsamples were soaked in 2.5% HCl solution for 6 hours to remove any carbonates, rinsed 3 to 5 times with demineralized water to bring the pH to around 6, centrifuged to remove excess water and then freeze-dried. Then, 5 to 10 mg of sediment was weighed into 8 x 5 mm tin capsules (Lüdi Swiss AG, Switzerland) and analyzed for δ^13^C values using an Elementar Vario EL Cube or Micro Cube elemental analyzer (Elementar Analysensysteme GmbH, Germany) interfaced to a PDZ Europa 20–20 isotope ratio mass spectrometer (Sercon Ltd., UK) at UC Davis Stable Isotope Facility (USA). The analytical precision, expressed as one standard deviation based on the results from multiple (n ≥ 4) analyses of a laboratory standard (nylon, bovine liver, glutamic acid, and peach leaves), was better than 0.2 ‰ for δ^13^C values, 2.6% for carbon content, and 0.3% for nitrogen content.

### Invertebrate remains

For the analysis of invertebrate remains, all sediment samples were deflocculated in 10% KOH for 2 hours at room temperature and sieved with a 100 μm mesh size. The sieve residue was then exposed for 20 hours to a 2M NH_4_Cl solution buffered with 0.35 NaOH to remove carbonates at neutral pH [46,47]. Different invertebrate remains were separated under a binocular microscope (magnification 20–50 x) into the following categories: ephippia of the cladoceran genera *Daphnia* and *Ceriodaphnia* (identification following [48]), head capsules of the chironomid groups Chironomini, Orthocladiinae, and Tanypodinae (identification following [49]), statoblasts of the Bryozoa genera *Plumatella* and *Cristatella* (identification following [50]), and *Chaoborus* and Ephemeroptera mandibles (identification following [49]). The total sum of the picked invertebrate remains was higher than 25 for all the samples. The relative abundance of the remains of these invertebrate taxa were plotted to provide supplementary information for our reconstruction. *Daphnia* ephippia and *Plumatella* statoblasts, which were found in sufficient quantities to measure δ^13^C values, were carefully cleaned with fine forceps and picked into silver cups (6 x 4 mm; Säntis, Switzerland). These remains were analyzed for δ^13^C values using a Costech ESC 4010 elemental analyzer interfaced via a ThermoConflo III to a Thermo Delta V isotope ratio mass spectrometer (IRMS) at the Alaska Stable Isotope Facility, University of Alaska, Fairbanks (USA). The analytical precision for δ^13^C values, expressed as one standard deviation based on the results from multiple (n ≥ 9) analyses of a laboratory standard (peptone), was better than 0.2 ‰. Isotopic data are reported as δ values in per mill relative to the VPDB standard (Vienna Pee Dee Belemnite).

### Land-use history

Past changes in vegetation and land use around Mekkojärvi were constrained using aerial images, historical documents, and pollen and charcoal analysis of the sediment record. Aerial images are available from 1949 A.D. [51] and historical documents, including maps and land tenure contracts from the end of the 19^th^ century [52,53]. Pollen and microscopic charcoal were analyzed from a total of 15 samples from the uppermost 32 cm of the record. Freeze-dried sediment was subsampled by weight to correspond to 1 cm^3^ wet volume and treated with HCl, KOH, HF, and acetolysis following standard methods [54]. *Lycopodium* tablets were added to the samples prior to chemical treatment, for estimating microscopic charcoal, spore and pollen concentrations [55]. Pollen and spores were identified using palynological keys and photo atlases [54,56,57]. Forty-seven pollen types were identified, and a pollen sum of higher than 500 was reached, except in the uppermost sample between 0 and 2 cm depth. Zonation of the pollen diagram was based on a constrained hierarchical clustering as implemented in the R package *rioja*, based on the CONISS algorithm [58], Euclidean distance as dissimilarity metric, and percentage data of tree, shrub, and herb pollen. The significance of zones was tested using the broken stick-model [59], and only significant zones are presented in the pollen diagram. Microscopic charcoal particles larger than 10 μm were counted following previously published protocols [60,61].

## Results

### Dating and geochemical properties

**Fig 2:**
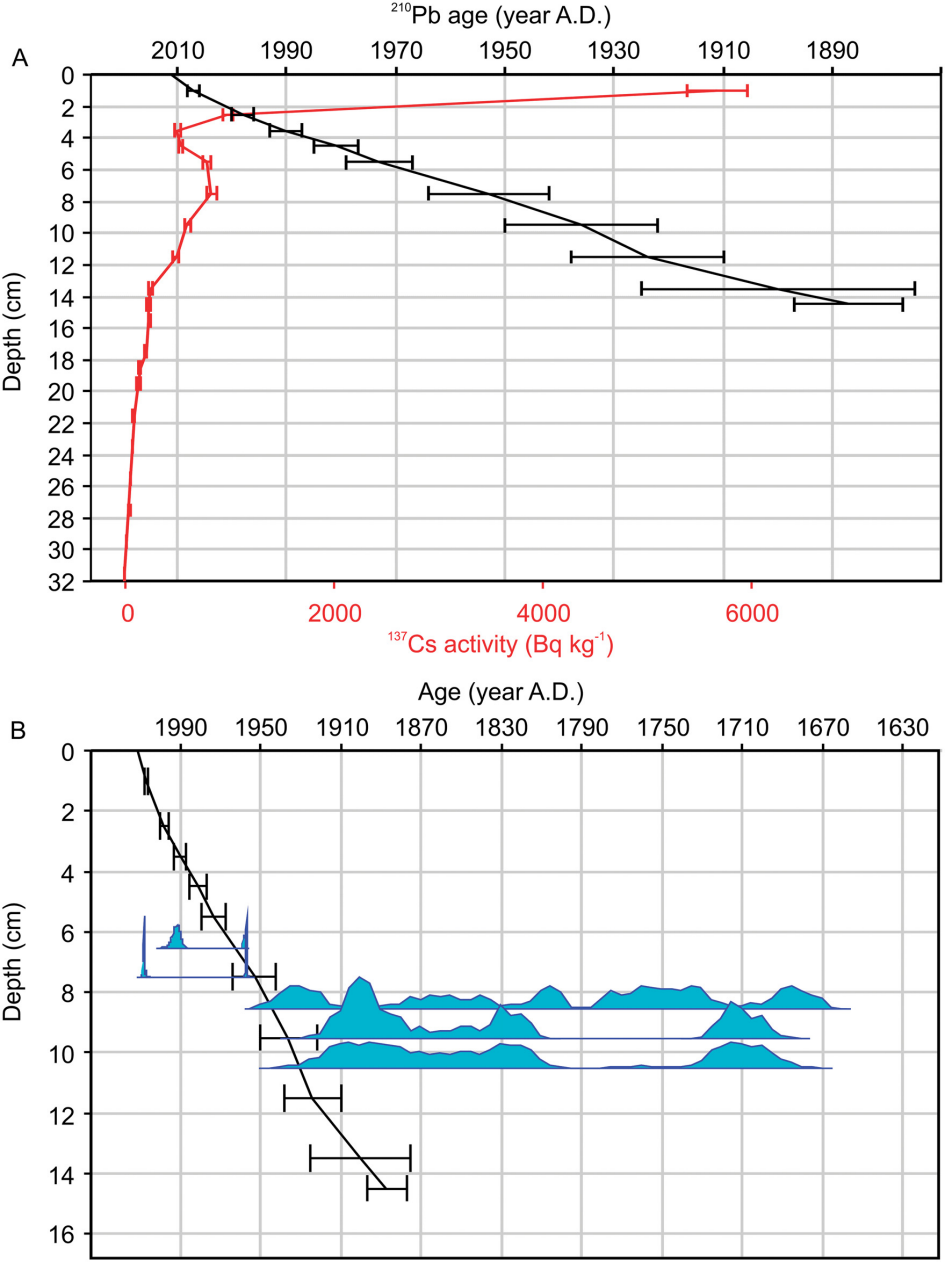
Dating of the Mekkojärvi sediment record. (A) Age-depth model based on a constant rate of supply model for ^210^Pb (black line, uncertainties represent one standard deviation) and ^137^Cs activity for comparison (red line, not used for the age model). (B) Probability distributions of ^14^C dates. X-axis represents ^210^Pb and calibrated ^14^C ages. Only the five uppermost ^14^C dates in agreement with ^210^Pb (black line) are presented.

Significant unsupported ^210^Pb activity was registered only down to a sediment depth of 14 to 15 cm (Fig 2). ^137^Cs analysis revealed the highest activity in the uppermost 0 to 2 cm sediment layer, instead of two maxima at 1963 and 1986 as are typically measured in European lakes, coinciding with the peak in above-ground nuclear bomb testing and the Chernobyl reactor accident [41]. A peak of ^137^Cs at a sediment depth from 5 to 8 cm that may correspond with the bomb testing in the 1960s agrees reasonably well with the ^210^Pb dating but is broader than expected. Similarly unusual ^137^Cs profiles have been regularly reported from soft-water lakes and can be related to delayed release of ^137^Cs from the catchment and vertical mobility of ^137^C due to release by decomposition and scarcity of clay minerals for adsorption [62,63]. One of the six ^14^C samples analyzed in the uppermost sediment layers indicated a ^14^C age significantly younger than the ^210^Pb-based estimates and therefore does not represent the age of the sediment matrix (S1 Table). This strongly suggests that the plant material was redeposited to older layers as may occur irregularly during lake sediment coring. However, the remaining five ^14^C dates were in agreement with the ^210^Pb dating and indicated that the increase of ^137^Cs preceding the local maximum at the sediment depth from 5 to 8 cm coincided with the expected increase in atmospheric ^14^C. This confirms that this ^137^Cs maximum represents the 1963 bomb peak and provides a reliable age constraint for our record. The five ^14^C-dated terrestrial macrofossil samples from the depth of 45 cm downwards returned unexpectedly old ages (the youngest age from 2600 ^14^C uncal. BP) and partially in inverse order (S1 Table). We attribute these old dates to remobilizing of old terrestrial plant material caused by peat removal during local forest use, which has been practiced in the area since at least the 16^th^ century [64,65]. The ^14^C dates in the lower part of the core are therefore not considered reliable.

**Fig 3:**
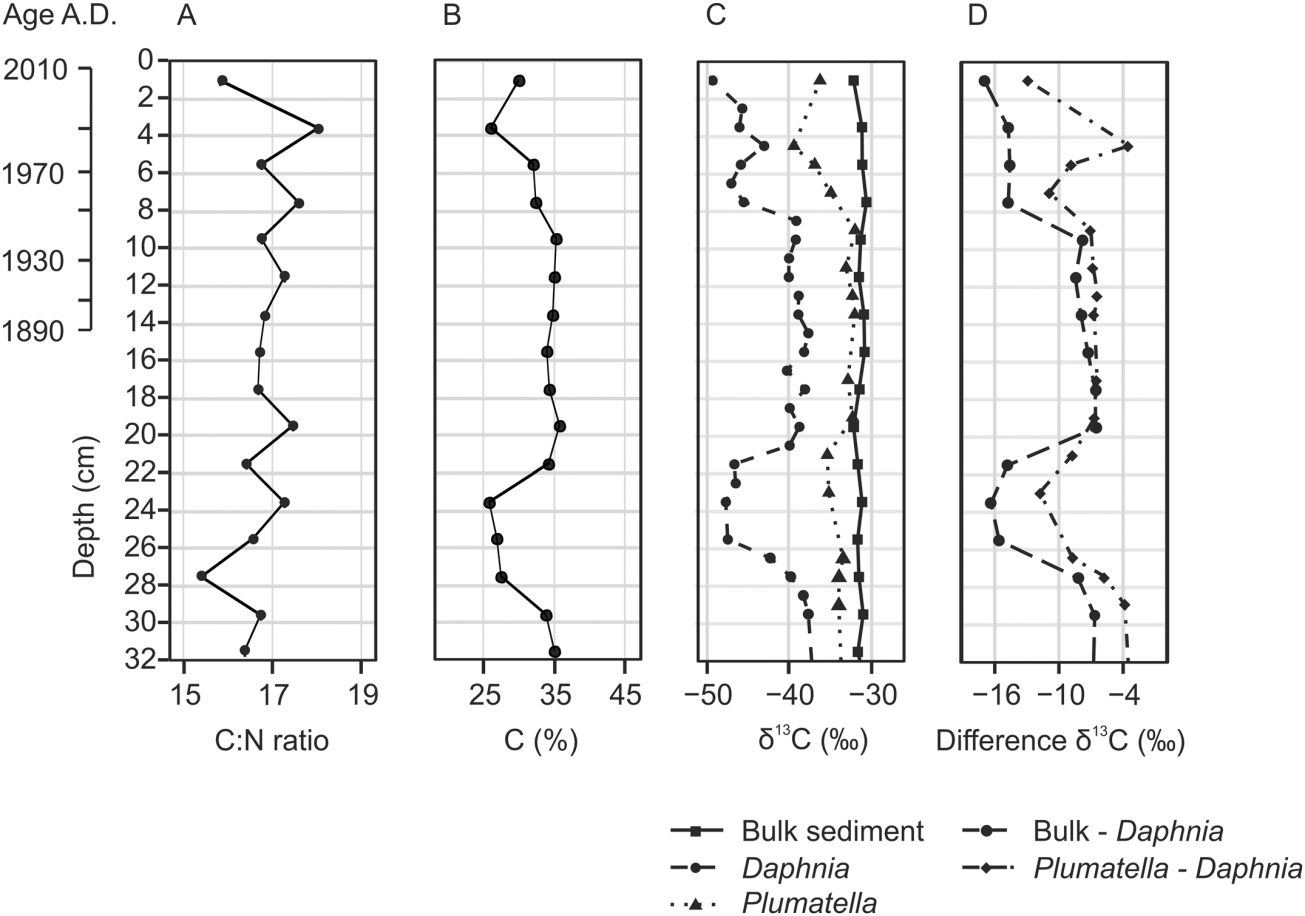
Sediment geochemical properties and δ^13^C values of invertebrate remains in the upper part of the Mekkojärvi sediment record. (A) Bulk sediment C:N ratio. (B) Bulk sediment organic carbon content. (C) δ^13^C values of bulk sediment, *Daphnia* ephippia and *Plumatella* statoblasts. (D) The difference between *Plumatella* statoblast and *Daphnia* ephippia δ^13^C values and between bulk sediment and *Daphnia* ephippia δ^13^C values. The age based on ^210^Pb dating is indicated on a separate scale bar.

The C:N ratio of SOM varied between 15 and 18 and carbon content between 25 and 36% (Fig 3). Three distinct changes in the carbon content were observed: between 27 and 30 cm, it decreased from 34 to 27%; between 24 and 21 cm, an abrupt increase from 27 to 34% was observed; and between 6 and 3 cm, carbon content decreased again from 34 to 26%.

### Invertebrate assemblages and δ^13^C values

**Fig 4:**
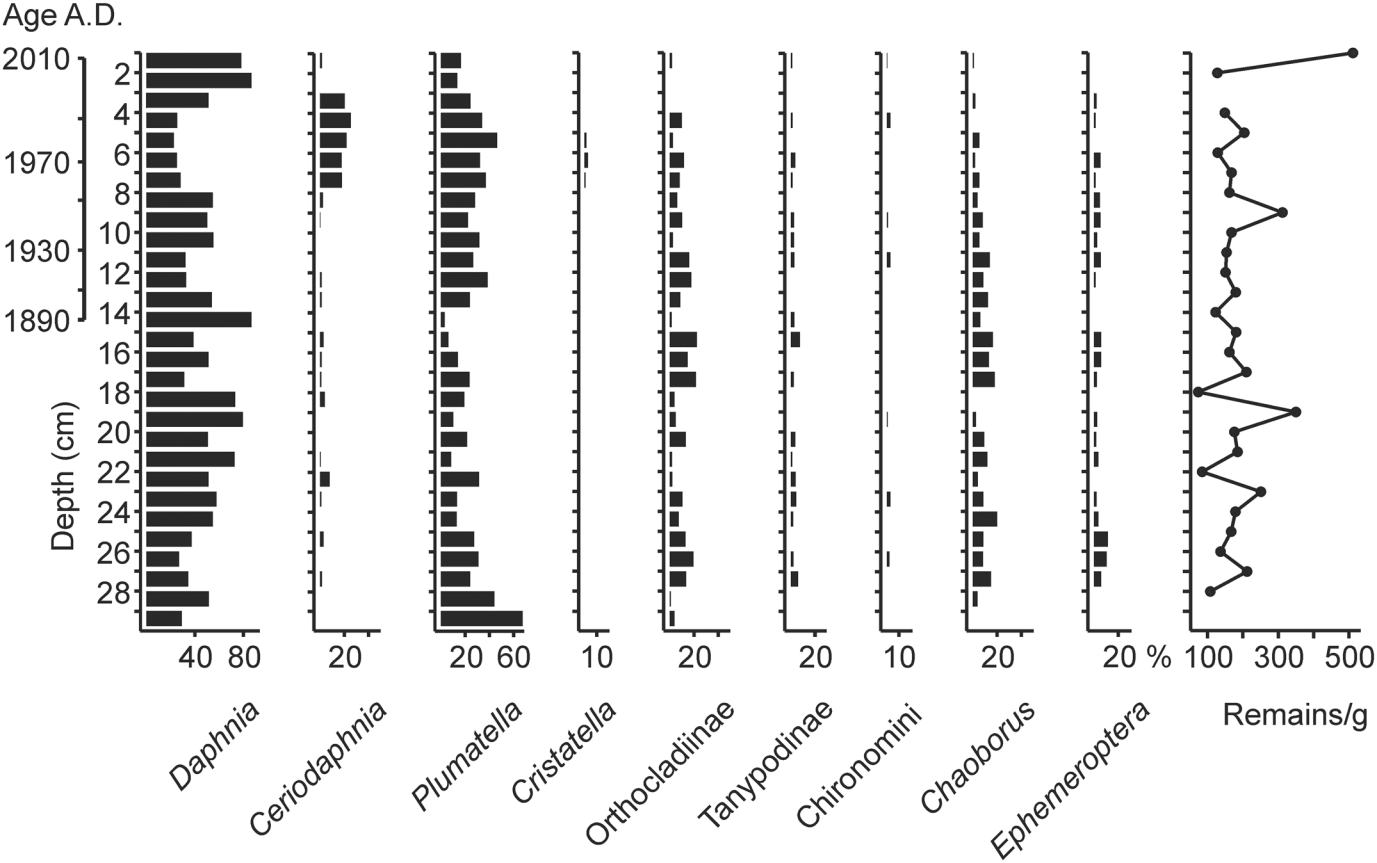
Invertebrate assemblages in the upper part of the Mekkojärvi sediment record. Percentage of the remains of selected invertebrate taxa relative to the total number of picked remains (size fraction > 100 μm) and number of picked remains per gram of dry sediment plotted against sediment depth are shown. The age based on ^210^Pb dating is indicated on a separate scale bar. No information on sediment weight for the depths 3 to 4 cm and 29 to 30 cm is available.

No major changes in the invertebrate assemblages were observed in the uppermost 30 cm of the Mekkojärvi sediment record (Fig 4). *Daphnia* ephippia were abundant throughout the record and comprised 20 to 90% of all invertebrate remains isolated in the size fraction larger than 100 μm. *Plumatella* statoblasts were also very abundant. The fraction of *Ceriodaphnia* ephippia was higher in the sediment layers from 3 to 8 cm than in the rest of the record. The abundance of Chironomidae head capsules was very low (0–30%) throughout the record. Almost no remains of Chironomidae, *Chaoborus*, and Ephemeroptera were found in the uppermost sediment layers (0–3 cm).

The δ^13^C values of bulk organic matter in the sediment were relatively constant at approximately -31 ‰ in the uppermost 30 cm (Fig 3). For *Daphnia* ephippia, the minimum sample weight for δ^13^C analysis was reached at 1 cm resolution (except the uppermost 2 cm) but for *Plumatella* statoblasts some samples had to be combined to a sample resolution of 2 cm in parts of the record. The δ^13^C values of *Daphnia* ephippia showed significant variability with values ranging from -49 to -36 ‰. In the uppermost 8 cm and at 27 to 21 cm depth, the δ^13^C values of *Daphnia* were lower than -45 ‰. From a sediment depth of 21 cm to 8 cm and in the layers deeper than 27 cm, the δ^13^C values of *Daphnia* ephippia were higher than -40h ‰. The δ^13^C values of *Plumatella* statoblasts showed some variations synchronous with those of *Daphnia* but of smaller amplitude with values between -39 and -32 ‰.

### Land-use history

**Fig 5:**
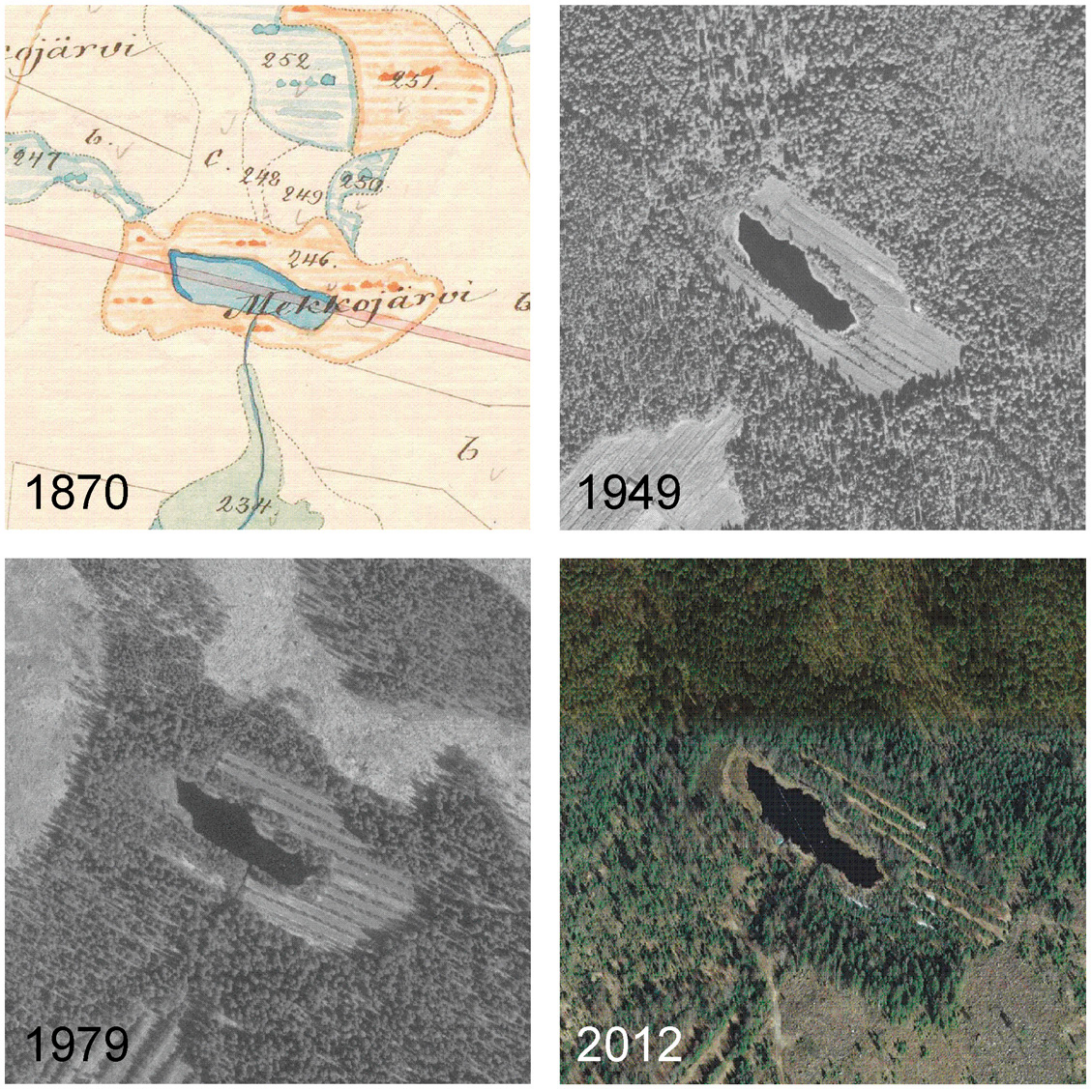
Historical map and aerial images showing the land-use changes in the catchment of Mekkojärvi. In the map from 1870, the shores of Mekkojärvi are categorized as wet meadow. The gradual afforestation in the catchment is shown in the aerial images from 1949, 1979, and 2012. Sources: The National Archives of Finland, Metsähallituksen historialliset kartat, Karta öfver Savijärvi block af Evois kronopark i Lampis socken och Tavastehus län (25S 01/07) and National Land Survey of Finland, Aerial photographs 04/2014. The map is reprinted from The National Archives of Finland under a CC BY license, with permission from The National Archives of Finland, original copyright 2014. The aerial images are reprinted from National Land Survey of Finland under a CC BY license in accordance with the National Land Survey open data license, version 1.0, 1 May 2012.

At present, Mekkojärvi is surrounded by forest with birch (*Betula alba*), spruce (*Picea abies*), and Scots pine (*Pinus sylvestris*). The old aerial images reveal that in the first half of the 20^th^ century a part of the catchment was tilled (Fig 5). The evidence of growing shrub or tree vegetation in the earliest aerial image from 1949, apparent as lines of trees in later images, indicates that cultivation in the field around Mekkojärvi must have been terminated shortly before 1949. According to historical land tenure contracts, the naturally wet meadow around the shores of Mekkojärvi (Fig 5) was drained and prepared for crop cultivation between 1914 and 1920 [53]. However, the tenure farm of Savijärvi situated close to the lake (Fig 1) was already occupied at the beginning of the 19^th^ century [53], and the meadow around Mekkojärvi was likely used for winter fodder production and grazing already before the tillage [53,64]. Land tenure contracts contain a reference to sowing with rye and other cereals in the meadows of the Savijärvi farm in 1888 and to grazing of livestock on the Mekkojärvi meadow in 1898 [53]. Before that, no documentation on the land use in the Mekkojärvi catchment is available. The Evo crown park was founded and forest guarding started in the mid-19^th^ century but before that the forests in the region were freely used for fuel collection, logging, tar production, and slash-and-burn cultivation at least since the 16^th^ century [64,65]. A large forest fire destroyed forests in the region in 1826 [65].

**Fig 6:**
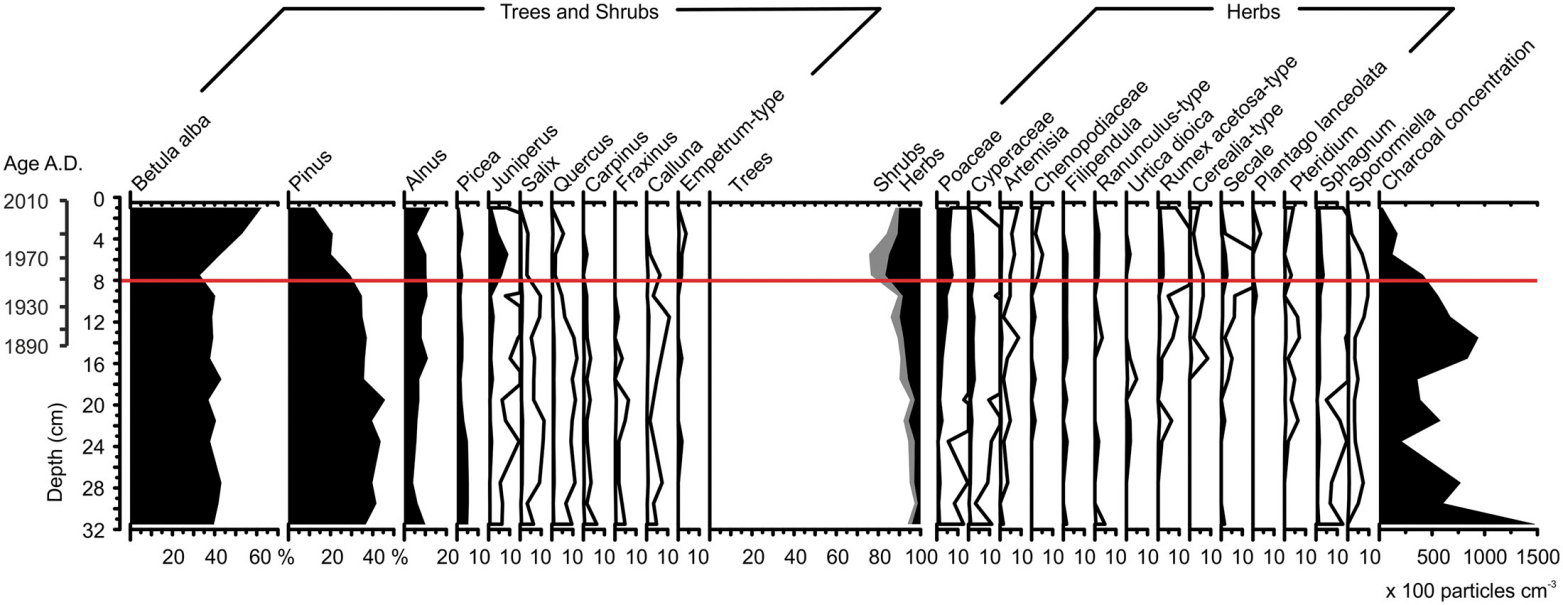
Pollen diagram of the upper part of the Mekkojärvi sediment record. The pollen spectra are plotted as percentages and charcoal as concentration against depth, age based on ^210^Pb dating is indicated on a separate scale bar. 10 × exaggeration is used to show changes in low abundance taxa, the horizontal line indicates the only statistically significant zonal boundary in the pollen data as assessed by constrained hierarchical clustering.

The land-use changes in the Mekkojärvi catchment are supported by our pollen analyses (Fig 6). The oldest analyzed part of the record (approx. 32–21 cm) is characterized by a low proportion of shrub and herb pollen, indicating a fairly closed forest and a low level of human disturbance in the catchment. However, relatively high charcoal concentrations in the sediment layers from 32 to 27 cm suggest local human activities (e.g. slash-and-burn cultivation). A second phase (approx. 21–8 cm) is characterized by gradually intensifying land use in the area, indicated by an increase of pollen of rye (*Secale*) and other cereals (Cerealia), weeds (*Artemisia*, *Urtica*, *Rumex*), typical pasture indicators (*Juniperus*, *Ranunculus*) [64], and charcoal, and by the decrease of pollen of trees such as *Picea* and *Pinus*. The third phase starts at approx. 8 cm sediment depth with a distinct increase of *Juniperus* followed by a rise in the pioneer tree species *Betula alba*, indicating early stages of forest succession leading to the forestation of the field around the lake. The relatively high percentages of herb pollen and anthropogenic indicators coinciding with this increase in *Juniperus* may be a consequence of reduced mowing and higher pollen release of meadow herb species. Decreasing charcoal concentrations in the uppermost sediment layers are in good agreement with a decrease in local anthropogenic activities in the vicinity of Mekkojärvi from approx. 8 cm onwards. The only significant zone boundary in the pollen record was identified at 8 cm sediment depth, coinciding with the increase in *Juniperus* pollen and the start of forestation of the catchment.

## Discussion

The very negative δ^13^C values of *Daphnia* ephippia in the surface sediments of Mekkojärvi (as negative as -49 ‰, Fig 3) are in line with previous studies suggesting high incorporation of methanogenic carbon in *Daphnia* diet in the contemporary ecosystem [7]. At present, pelagic primary production in Mekkojärvi is very low throughout the year (mostly below 100 mg C m^-2^ day^-1^) [7,35], and phytoplankton contributes only between 25 and 71% to *Daphnia* diet, depending on the season [7]. With such low algal availability in the epilimnion, *Daphnia* may migrate vertically and exploit microbial food resources, including MOB, in the oxic-anoxic boundary layer or even deeper in the anoxic hypolimnion [31,66]. The less negative δ^13^C values of *Plumatella* than those of *Daphnia* might be explained by the different mobility of these planktivorous organism groups. Although *Plumatella* can also ingest microbial biomass, it is sessile and can only feed on particles that reach its location [32,67]. Therefore, δ^13^C values of *Plumatella* are generally expected to reflect more strongly those of phytoplankton and not to be as negative as those of *Daphnia* [68,69].

Our δ^13^C analysis in the Mekkojärvi sediment record revealed that major shifts in the δ^13^C values of *Daphnia* have occurred in the past (Fig 3). The modern situation in the lake with exceptionally negative δ^13^C values of zooplankton only developed in the 1950s, although similar values were also observed in some older sediment layers (26–21 cm). Assuming sedimentation rates similar to the uppermost layers, these sediments would represent the beginning of the 19^th^ century. From a sediment depth of 21 cm (estimated to originate from around the 1830s) to the late 1940s and in the sediment layers deeper than 27 cm (estimated to originate from the 18^th^ century), the δ^13^C values of *Daphnia* ephippia are approximately 10 ‰ higher than the modern values. This large amplitude of variations can only be explained by major changes in the carbon pathways in the lake. Since δ^13^C values as low as -50 ‰ are only possible if a substantial amount of carbon in the diet of *Daphnia* originates from CH_4_ [7], reduced proportion of methanogenic carbon in the *Daphnia* diet is the most plausible explanation for the distinctly higher δ^13^C values during some periods. This can be produced by mechanisms which either reduce the CH_4_ availability for MOB and therefore the growth rates and biomass of MOB in the water column, or by mechanisms which increase the availability of phytoplankton or other organic matter sources with δ^13^C values higher than those of MOB. The δ^13^C values of *Daphnia* ephippia in the sediments could also be influenced by seasonal timing of ephippia production. However, the variation up to 10 ‰ in the δ^13^C values of *Daphnia* ephippia is very large compared to the observed seasonal variation in the δ^13^C values of *Daphnia* in Mekkojärvi (7.7 ‰) [5] and would require a seasonal shift of the entire ephippium production. In principle, this could be caused by species shifts in *Daphnia* assemblages, since some *Daphnia* species tend to produce their ephippia in spring and early summer rather than late summer and autumn [70]. However, we regard major species shifts very unlikely since no changes in *Daphnia* ephippia morphology were observed in the sediment record. Furthermore, shifts in timing of ephippia production or in *Daphnia* assemblage cannot explain the synchronous, although less pronounced shifts in δ^13^C values of *Plumatella* (approx. 4 ‰) observed in the record.

**Fig 7:**
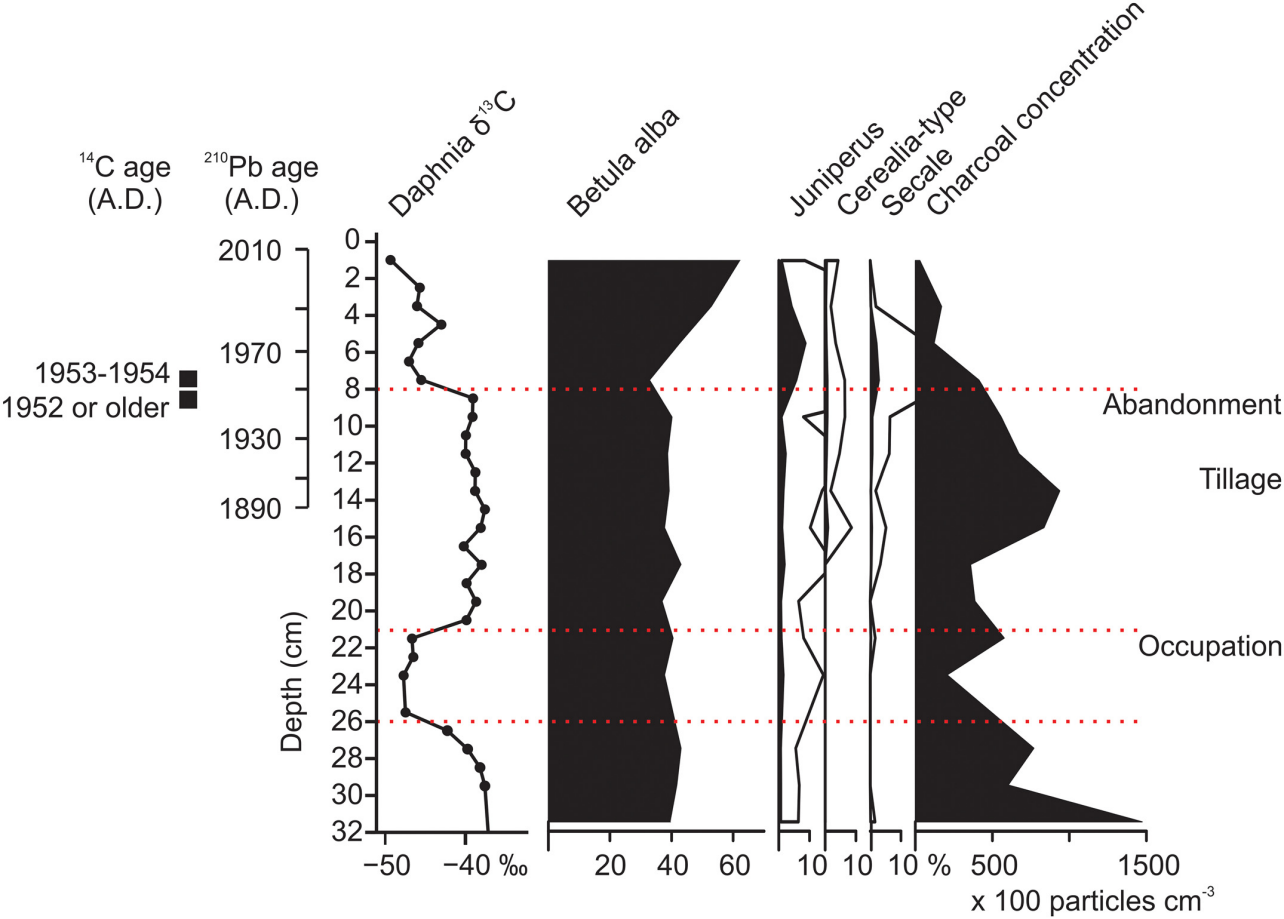
δ^13^C values of *Daphnia* ephippia relative to the most relevant pollen indicators, charcoal, and historical events in the catchment. The main events include occupation of the Savijärvi farm in the beginning of the 19^th^ century, drainage and tillage of Mekkojärvi meadow in the 1910s, and abandonment of the tillage in the 1940s. The age based on ^210^Pb dating on a separate scale bar and the calibrated ^14^C dates in the sediment depths from 7 to 9 cm are indicated. Since the age of sediments older than 1950 is poorly constrained, the historical events are drawn in the diagram at approximate locations based on modern sediment accumulation rates and the presented interpretation of sediment record.

Interestingly, the uppermost shift in the δ^13^C values of *Daphnia* and *Plumatella* at 8 cm coincides with the abandonment of the tillage around the lake and the beginning of forestation, as indicated e.g. by the distinct increase in juniper pollen in the sediments (Fig 7). The earlier increase in δ^13^C values between 21 and 20 cm depth (estimated age 1830s to 1840s) corresponds to the first indications of cereals (*Secale*) in the record, although evidence of human-induced land-use changes are not as clear in this part of the record as in the uppermost sediment layers. Agricultural land use around Mekkojärvi from the beginning of the 19^th^ century to the 1940s could have reduced the availability of MOB for planktivorous invertebrates. Deforestation can promote more frequent or deeper wind-induced mixing of the water column and therefore reduce the volume of the anoxic hypolimnion, where CH_4_ accumulates, which in turn can lead to lower MOB production and lower incorporation of methanogenic carbon by *Daphnia* during the autumnal turn over, when a large part of the ephippia is usually produced [70]. Alternatively, agricultural land use could have increased the availability of phytoplankton to planktivorous invertebrates. Generally, intensified human activity in lake catchments leads to higher nutrient inputs to lakes due to increased soil erosion, manuring, biomass burning, and logging, and consequently to higher primary production [13,71]. If phases of intensified land use in the catchment were associated with higher phytoplankton biomass in the lake, *Daphnia* could have relied more on phytoplankton as a food source. Furthermore, higher δ^13^C values of phytoplankton during phases of intensified land use may have affected the δ^13^C values of planktivorous invertebrates [72]. More productive conditions can lead to lower DIC concentrations during active phytoplankton growth and hence increased δ^13^C values of phytoplankton [73,74].

Indicators for oxic conditions (e.g. Ephemeroptera) [75] or for anoxic conditions (e.g. *Chaoborus*) [76] in the invertebrate assemblage do not show major changes in the sediment record (Fig 4). However, this does not necessarily mean that no changes in oxygenation took place during the phases of intensified land use between the 1830s and the 1940s. Ephemeroptera remains are very rare in all samples examined from the record. In small lakes such as Mekkojärvi, they may have been transported from the nearshore areas of the lakes and might not indicate conditions in the deepest area of the lake basin. Furthermore, *Chaoborus* abundances in fishless lakes are less strongly influenced by oxygen conditions than in lakes with intensive fish predation [77]. Similarly, no decrease in the C:N ratio of the SOM, which can reflect increased primary production in lakes [22], is evident in the sediment layers originating from the period of intensified land use (Fig 3). However, the SOM in Mekkojärvi is strongly dominated by terrestrial material (C:N ratio > 15), which means that a very substantial increase in autochthonous production would be needed to change the C:N ratio of the SOM. Furthermore, changes in the C:N ratio during the period of intensified land use may have been compensated by increased terrestrial inputs due to draining of the organic catchment soils, as suggested by increasing organic carbon content of sediment layers originating from the period of intensified land use (Fig 3). If variations in the δ^13^C values of phytoplankton were the main process responsible for the changes in the δ^13^C values of planktivorous invertebrates, it would be expected that *Daphnia* and *Plumatella* would show shifts of similar amplitude. This is clearly not the case (Fig 3). It is therefore apparent that the mechanisms increasing the δ^13^C values of planktivorous invertebrates during the intensified land use are mainly related to variations in the amount of available organic matter produced by phytoplankton or of MOB and not only to changes in the δ^13^C values of the phytoplankton. Since the most recent shift in the δ^13^C values of *Daphnia* from approximately -39 ‰ to -47 ‰ occurred immediately after the abandonment of the tillage in the 1950s, when no high vegetation had yet developed to shelter the lake (Fig 5), it is also clear that changes in wind-induced water mixing were probably not the only factor leading to dietary shifts of *Daphnia*.

The earlier shifts in δ^13^C values of *Daphnia* and *Plumatella* observed in the deeper sediment layers (27–25 cm; approximately late 18^th^ or early 19^th^ century) cannot be firmly linked to historical land-use changes because no historical sources for land use in the Mekkojärvi catchment are available for this period. Also, the pollen record shows no evidence for major changes in regional vegetation at this depth (Fig 6), although changes in local forest use often do not lead to noticeable variations in the abundance of tree pollen in the sediment record of lakes in forested landscapes [78]. However, the increased concentration of charcoal in pollen samples in the sediment layers from 32 to 27 cm suggest local land use (e.g. slash-and-burn cultivation) that occurred in the catchment during the time represented by the deepest layers of our sediment record. Old and partially inverse ages of the five terrestrial macrofossil samples from the depth 45 cm downwards strongly suggests that the lower part of the sediment record has received input of old, reworked terrestrial material from the catchment indicating peat removal and supporting local forest use.

## Conclusions

Our study shows that the incorporation of methanogenic carbon into the food web in small boreal lakes can vary significantly on multi-decadal time scales. For Mekkojärvi, we demonstrated at least three major shifts in the carbon pathways of the pelagic food web within the past centuries, and that the lake only recently (1950s) obtained its present state with high contributions of methanogenic carbon to the pelagic food web. Although we cannot constrain the exact in-lake mechanisms leading to the observed shifts, our analyses indicate that they are associated with changes in forest and land use in the catchment. The most recent shift in carbon sources for the planktonic food web coincides closely with the land-use changes which led to a forestation of the lake catchment. For earlier shifts the relationship with land-use changes is less clear, but the increase in the δ^13^C values of *Daphnia* at 20.5 cm depth coincides with a phase of increasing anthropogenic indicators in the pollen record suggesting that these earlier changes in the carbon sources for the planktonic food web may also have been related to variations in land use in the catchment. Due to our inability to date the lowermost sediment layers and to demonstrate that they are older than the forest use in the region, it remains unclear whether the present state of the lake, with high contributions of methanogenic carbon to the pelagic food web, represents the original, pre-impact state of the lake.

At Mekkojärvi, land-use changes in the catchment occurred on a small spatial scale and the absolute changes in nutrient inputs, lake productivity, or oxygen concentrations were probably relatively minor. Nevertheless, they caused significant variations in the contribution of different carbon sources to the pelagic food web, at least in case of the youngest recorded shift in in-lake carbon cycling. This sensitivity in the carbon cycling of small boreal lakes has important implications for their protection and for conservation strategies for small lakes in forested landscapes.

Our study provides an example of how changes in the carbon cycling in lakes may be overlooked in conventional biomonitoring, palaeoecological, or sedimentological studies [79,80]. Modern ecosystem studies defining "natural" reference conditions are often based on assessments of the taxonomic composition of invertebrate or algal assemblages [11]. We were not able to identify invertebrate remains in the sediments of Mekkojärvi to species or species group level. Nevertheless, our study indicates that, at the family- to genus-level identification we achieved, major alterations in carbon pathways may not be apparent in the abundance of individual invertebrate groups or in the overall composition of invertebrate assemblages in lakes (Fig 4). Similarly, δ^13^C values or C:N ratios of the sediment did not record variations which clearly coincided with the observed shifts in the δ^13^C values of planktivorous invertebrate remains in Mekkojärvi. For studies assessing natural reference conditions and pre-impact states of lake ecosystems, the inclusion of stable carbon isotope analyses of aquatic invertebrates and their remains in the sediments may help to detect variations in the relevance of different carbon pathways within and between lakes that may otherwise be difficult to observe.

## Supporting Information

S1 Table Radiocarbon dates of terrestrial plant macrofossils for the Mekkojärvi sediment core. For the six uppermost samples, 95% confidence intervals for the calibrated ^14^C dates are provided, for comparison together with 95% age confidence intervals based on the ^210^Pb model (instead of the conventionally used one standard deviation intervals as shown in Fig 2). Calibrated ages are presented in year AD at 2σ range with cumulative probability in %. Parts of the probability distribution which are consistent with the ^210^Pb dating are highlighted in bold. ^210^Pb ages for the depths 6.5, 8.5, and 10.5 cm are interpolated.
(DOCX)
