# Supplementary material for: Land Use Affects Carbon Sources to the Pelagic Food Web in a Small Boreal Lake
Source: PLoS One. 2016 Aug 3;11(8):e0159900. doi: 10.1371/journal.pone.0159900 (PMC4972442; doi:10.1371/journal.pone.0159900)
Supplement: S1 Table — For the six uppermost samples, 95% confidence intervals for the calibrated 14C dates are provided, for comparison together with 95% age confidence intervals based on the 210Pb model (instead of the conventionally used one standard deviation intervals as shown in Fig 2). Calibrated ages are presented in year AD at 2σ range with cumulative probability in %. Parts of the probability distribution which are consistent with the 210Pb dating are highlighted in bold. 210Pb ages for the depths 6.5, 8.5, and 10.5 cm are interpolated. (DOCX) [file pone.0159900.s001.docx]

**S1 Table. Radiocarbon dates of terrestrial plant macrofossils for the Mekkojärvi sediment core.**

| **Depth**  **(cm)** | **Laboratory code** | **Uncalibrated ^14^C age**  **(year BP ± 1σ)** | **Calibrated ^14^C age**  **(year AD with 95% conf.)** | **^210^Pb age**  **(year AD with 95% conf.)** | **Remarks** |
| --- | --- | --- | --- | --- | --- |
| 6.5 | BE-4872.1.1 | -1095 ± 114 | **1958 (6%) 1959**  1988 (89%) 1997 | 1946 (95%) 1980 |  |
| 7.5 | BE-4873.1.1 | -348 ± 41 | **1957 (6%) 1958**  2008 (89%) 2012 | 1931 (95%) 1976 |  |
| 8.5 | BE-4874.1.1 | 149 ± 25 | 1668 (16%) 1707  1719 (33%) 1781  1797 (11%) 1825  1832 (17%) 1885  **1913 (17%) 1949**  **1954 (1%) 1955** | 1920 (95%) 1970 |  |
| 9.5 | BE-4875.1.1 | 66 ± 25 | 1695 (23%) 1726  1813 (17%) 1839  1841 (5%) 1854  1857 (1%) 1861  **1867 (49%) 1917** | 1909 (95%) 1964 |  |
| 10.5 | BE-4876.1.1 | 86 ± 31 | 1685 (26%) 1731  **1808 (69%) 1927** | 1903 (95%) 1958 |  |
| 11.5 | BE-4877.1.1 | -606 ± 25 | 1958 (3%) 1958  2001 (92%) 2005 | 1896 (95%) 1952 | ^14^C date completely discarded due to the unreasonable young age compared to the sediment |
| 45.5 | Poz-55407 | 3765 ± 35 |  |  | Discarded due to the unreasonable old age |
| 56.5 | Poz-55408 | 2640 ± 30 |  |  | Discarded due to the unreasonable old age |
| 56.5 | Poz-56346 | 2670 ± 40 |  |  | Discarded due to the unreasonable old age |
| 60.5 | Poz-55410 | 4200 ± 35 |  |  | Discarded due to the unreasonable old age |
| 60.5 | Poz-55798 | 4165 ± 35 |  |  | Discarded due to the unreasonable old age |
| 69.5 | Poz-54357 | 7310 ± 50 |  |  | Discarded due to the unreasonable old age |
| 76.5 | Poz-54358 | 5580 ± 50 |  |  | Discarded due to the unreasonable old age |

For the six uppermost samples, 95 % confidence intervals for the calibrated 14C dates are provided, for comparison together with 95 % age confidence intervals based on the 210Pb model (instead of the conventionally used one standard deviation intervals as shown in Fig 2). Calibrated ages are presented in year AD at 2σ range with cumulative probability in %. Parts of the probability distribution which are consistent with the 210Pb dating are highlighted in bold. 210Pb ages for the depths 6.5, 8.5, and 10.5 cm are interpolated.
